# The Prevalence and Impact of Atrial Fibrillation on Patients with Chronic Total Occlusions: Insights from the National Inpatient Sample

**DOI:** 10.3390/jcdd12030100

**Published:** 2025-03-14

**Authors:** Maximilian Will, Konstantin Schwarz, Eric Holroyd, Josip A. Borovac, Adnan I. Qureshi, Gregory Y. H. Lip, Julia Mascherbauer, Gregor Leibundgut, Thomas W. Weiss, Chun Shing Kwok

**Affiliations:** 1 Karl Landsteiner University of Health Sciences, Dr. Karl-Dorrek-Straße 30, 3500 Krems, Austria; konstantin.schwarz@stpoelten.lknoe.at (K.S.);; 2 Division of Internal Medicine 3, University Hospital St. Pölten, Dunant-Platz 1, 3100 St. Pölten, Austria; 3 Karl Landsteiner Institute for Cardiometabolics, Karl Landsteiner Society, 3100 St. Pölten, Austria; ordination@doktorweiss.at; 4 Department of Cardiology, University Hospitals of North Midlands NHS Foundation Trust, Stoke-on-Trent ST4 6QG, UKshingkwok@doctors.org.uk (C.S.K.); 5 Department of Cardiology, Mid Cheshire Hospitals NHS Trust, Crewe CW1 4QJ, UK; 6 Division of Interventional Cardiology, Cardiovascular Diseases Department, University Hospital of Split, 21000 Split, Croatia; 7 Zeenat Qureshi Stroke Institute and Department of Neurology, University of Missouri, Columbia, MO 65201, USA; qureshai@gmail.com; 8 Liverpool Centre for Cardiovascular Science at University of Liverpool, Liverpool John Moores University, Liverpool Heart & Chest Hospital, Liverpool L17 6BD, UK; 9 Danish Center for Health Services Research, Department of Clinical Medicine, Aalborg University, 9220 Aalborg, Denmark; 10 Department of Cardiology, Basel University Hospital, 4031 Basel, Switzerland

**Keywords:** atrial fibrillation, chronic total occlusion, mortality, ischemic stroke, major bleeding

## Abstract

The impact of atrial fibrillation (AF) on patients with chronic total occlusions (CTOs) at the national level remains unclear. In this study, we conducted a retrospective analysis of data from the National Inpatient Sample to assess the characteristics and in-hospital outcomes of patients with CTO based on the presence or absence of AF. Multiple logistic and linear regressions examined factors associated with AF and evaluated its impact on length of stay (LoS), cost, and mortality. The analysis included 480,180 patients diagnosed with CTO, with AF present in 28.0% of cases. Patients with CTOs and AF were older (median age 73 vs. 66 years, *p* < 0.001) and exhibited lower female representation (25.0% vs. 27.9%, *p* < 0.001). Factors most strongly associated with AF included previous heart failure (OR 1.98, 95% CI 1.92–2.05, *p* < 0.001), liver disease (OR 1.37, 95% CI 1.27–1.48, *p* < 0.001), and obesity (OR 1.25, 95% CI 1.20–1.30, *p* < 0.001). AF correlated with increased in-hospital mortality (OR 1.29, 95% CI 1.18–1.40, *p* < 0.001), ischemic stroke (OR 1.27, 95% CI 1.13–1.42, *p* < 0.001), and major bleeding (OR 1.38, 95% CI 1.30–1.46). Moreover, AF was associated with a longer LoS (coef 1.58, 95% CI 1.50 to 1.67, *p* < 0.001) and higher in-hospital costs (coef 6.22, 95% CI 5.81 to 6.63, *p* < 0.001). Patients with CTOs and AF were older and had more underlying health problems compared to patients without AF. The patients with AF have worse outcomes in terms of mortality, ischemic stroke, major bleeding, length of stay, and costs.

## 1. Introduction

Atrial fibrillation (AF) is the most common arrhythmia worldwide and is known to exert a significant influence on cardiovascular morbidity and mortality [1]. Coronary artery disease (CAD) is the most common cardiovascular disease worldwide, and both AF and CAD have similar risk factors, which creates a bidirectional relationship between the two conditions that often occur together [2]. Additionally, CAD and AF have overlapping pathophysiological foundations [3].

A chronic total occlusion (CTO) is one of the most extreme manifestations of CAD and is common in patients with chronic coronary syndromes [4]. Several studies indicate that CTOs are associated with poor outcomes and higher mortality rates in different populations with coronary artery disease due to various underlying mechanisms [5,6]. Consequently, in some patients, particularly those who are symptomatic and experience angina despite medical therapy, there is evidence to support the use of revascularization techniques to open chronic total coronary occlusion if imaging methods demonstrate viability and ischemia in the territory supplied by the CTO vessel [7,8,9,10].

Recently, the negative impact of AF on mortality in patients undergoing CTO-PCI has been reported [11,12]. However, due to the complex nature of the intervention and lack of high-quality evidence regarding percutaneous coronary revascularization, many patients are treated conservatively [13]. The impact of AF on patients with CTOs, regardless of their treatment path has not been subject to much investigation. In this manuscript, we describe our retrospective analysis of data from the National Inpatient Sample to evaluate the population with a diagnosis of CTO and determine the factors associated with AF and the outcomes associated with AF compared to those without AF.

## 2. Methods

This manuscript was prepared in accordance with the recommendations of the STROBE criteria. Institutional review board approval for the study was not required for analysis of data from that National Inpatient Sample.

### 2.1. Study Design and Dataset

We conducted a retrospective cohort study by analyzing hospital admission data from the NIS between 2016 and 2020. The NIS is the largest all-payer inpatient care database in the United States, which is produced by the Healthcare Cost and Utilization Project (HCUP) at the Agency for Healthcare Research and Quality (AHRQ). The NIS hospital admission data were obtained from approximately 20% of the samples of hospitals in the United States, which translates to 5 to 8 million hospital admissions from approximately 1000 hospitals. It contains more than 100 clinical and non-clinical variables, including diagnostic codes, procedure codes, patient demographics, and patient admission and discharge status. The data from the NIS can be weighted to generate national estimates.

Hospital admissions with a diagnosis of CTO were included in the analysis based on the International Classification of Diseases, 10th Revision, Clinical Modification (ICD-10-CM) primary diagnosis codes to identify the hospital admissions where patients were diagnosed with coronary CTO (I25.82). Hospital admissions for patients with ages less than 18 years and those with missing values for age, sex, and in-hospital mortality were excluded.

### 2.2. Variable Definition

Clinical comorbidity variables for each admission were determined based on the ICD-10 codes or data available in the NIS, as defined in Supplementary Table S1. The primary exposure variable of interest was atrial fibrillation or flutter (I48*). Additional demographic variables, including age, sex, race, primary expected payer, and income based on ZIP code, were collected. Also, comorbidities such as smoking, alcohol misuse, obesity, hypertension, hyperlipidemia, diabetes mellitus, previous myocardial infarction, previous stroke, previous heart failure, previous venous thrombosis/embolism, chronic lung disease, chronic kidney disease, liver disease, anemia, any cancer, and dementia were determined. Hospital variables, including elective admission, weekend admission, season of admission, rural or urban designation of hospital, and hospital bed size, were extracted. The main outcome was in-hospital mortality, and the secondary outcomes were in-hospital acute ischemic stroke, major bleeding, length of stay, and in-hospital cost. In order to explore the impact of the primary diagnosis, we used the first digit of the primary or first ICD-10 code to define the category for the reason for admission. We compared the unadjusted, adjusted for all variables except the category of the primary diagnostic code, and adjusted for all variables including the category of the primary diagnostic code. As the majority of admissions were for diseases of the circulatory system, we further divided this category into the most common 3-digit diagnoses. As a sensitivity analysis, the cohort was divided according to the cause for hospitalization, and the proportion of patients with and without AF, as well as the mortality in each group, was determined. A further analysis was performed to identify the impact of AF on mortality for patients with a primary diagnosis of NSTEMI (ICD-10 code I21.4), NSTEMI (ICD-10 codes I21.0, I21.1, I21.2, I21.3), heart failure (I50), and for patients with heart failure who received intra-aortic balloon pump (ICD-10 code 5A02210) and left ventricular assist device (ICD-10 codes 5A02116, 5A0211D, 5A02216, 5A0221D).

### 2.3. Statistical Analysis

Statistical analysis was performed on STATA 13.0 (College Station, TX, USA). Descriptive statistics were presented for the patient characteristics, hospital characteristics and outcomes stratified by the presence or absence of AF. For continuous variables, the median and interquartile range were presented, and the median test on Stata was used to determine if there were statistical differences between the group with and without AF. Categorical variables were described with percentages, and a Chi^2^ test was used for determining if there were differences for the group with AF. Multiple logistic regression was performed to identify the independent predictors of AF in CTOs, with patient demographics, hospital, comorbidities, and severity markers as candidate variables in the adjusted model. Multiple logistic regressions were used to determine the impact of AF on in-hospital mortality, ischemic stroke, and major bleeding after adjustments for baseline variables. Multiple linear regression was used to estimate the impact of AF on length of stay and cost with adjustments for baseline variables. A sensitivity analysis was performed, evaluating the impact of AF on outcomes for the subgroup of patients with CTO who underwent PCI.

## 3. Results

**Figure 1:**
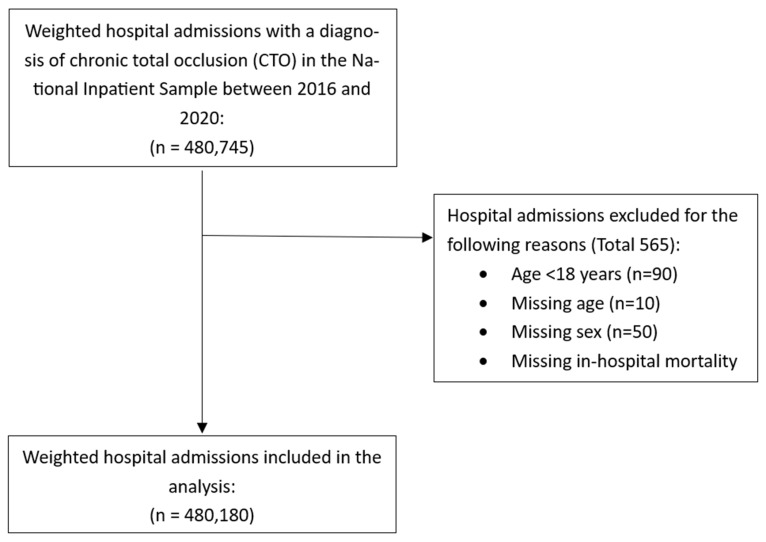
Flow diagram of identification of hospital admission with atrial fibrillation and chronic total occlusions.

The process of identification of hospital admissions for patients with CTO is shown in Figure 1. There were a total of 480,180 hospital admissions included in the analysis with CTO.

The characteristics of patients with CTOs were analyzed based on the presence or absence of atrial fibrillation. The results are presented in Table 1. There were 134,604 hospital admissions with AF (28.0%). The median age of patients with AF was significantly higher than those without AF (73 vs. 66 years, *p* < 0.001). Female representation was slightly lower in the AF group (25.0% vs. 27.9%, *p* < 0.001). Smoking and alcohol misuse was more prevalent in the group without AF compared to patients with AF (1.4% vs. 0.9%, *p* < 0.001 and 1.8% vs. 1.5%, *p* = 0.014, respectively). The prevalence of hypertension (90.7% vs. 86.9%, *p* < 0.001), previous myocardial infarction (31.5% vs. 29.6%, *p* < 0.001), previous stroke (14.1% vs. 11.5%, *p* < 0.001), previous heart failure (48.4% vs. 28.8%, *p* < 0.001), previous venous thromboembolism (5.0% vs. 3.9%, *p* < 0.001), chronic lung disease (31.4% vs. 24.7%, *p* < 0.001), chronic kidney disease (37.5% vs. 26.0%, *p* < 0.001), liver disease (5.1% vs. 3.9%, *p* < 0.001), anemia (10.2% vs. 7.8%, *p* < 0.001), and cancer (5.0% vs. 3.6%, *p* < 0.001) was higher in the AF group. Fewer patients had PCI (23.2% vs. 39.9%, *p* < 0.001).

In terms of crude outcomes, patients with AF had more major bleeding (9.7% vs. 6.2%, *p* < 0.001), ischemic stroke (2.0% vs. 1.5%, *p* < 0.001) and in-hospital mortality (4.4% vs. 2.9%, *p* < 0.001) compared to patients with CTO and no AF. The median hospital length of stay and in-hospital cost was also greater in the group with AF (5 vs. 3, *p* < 0.001 and USD 21,671 vs. USD 18,747, *p* < 0.001, respectively).

The results of the multiple logistic regression to evaluate the predictors of AF is shown in Table 2. Previous heart failure was the strongest factor associated with AF (OR 1.98 95% CI 1.92–2.05, *p* < 0.001). Other factors associated with increased odds of AF were liver disease (OR 1.37 95% CI 1.27–1.48, *p* < 0.001), obesity (OR 1.25 95% CI 1.20–1.30, *p* < 0.001), chronic kidney disease (OR 1.20 95% CI 1.16–1.24, *p* < 0.001), and hypertension (OR 1.17 95% CI 1.11–1.23, *p* < 0.001). Older age was associated with increased odds of AF (OR 1.05 95% CI 1.04–1.5, *p* < 0.001), but female sex was associated with reduced odds of AF (OR 0.75 95% CI 0.73–0.78, *p* < 0.001). Compared to white patients, patients who were black (OR 0.59 95% CI 0.55–0.62, *p* < 0.001), Native American (OR 0.60 95% CI 0.48–0.75, *p* < 0.001), and Hispanic (OR 0.62 95% CI 0.58–0.66, *p* < 0.001) were less likely to have AF.

Table 3 evaluates the odds of adverse outcomes and regression coefficients for length of stay and cost for AF in patients with CTO. AF was associated with an increase in in-hospital mortality (OR 1.29 95% CI 1.18–1.40, *p* < 0.001), ischemic stroke (OR 1.27 95% CI 1.13–1.42, *p* < 0.001), and major bleeding (OR 1.38 95% CI 1.30–1.46). It was also associated with longer length of stay (coef 1.58 days 95% CI 1.50 to 1.67, *p* < 0.001) and greater in-hospital cost (coef USD 6219 95% CI 5811 to 6627, *p* < 0.001). Sensitivity analysis for the subgroup of patients who underwent PCI showed even greater odds of in-hospital mortality (OR 1.55 95% CI 1.35–1.78, *p* < 0.001), ischemic stroke (OR 1.53 95% CI 1.19–1.96, *p* = 0.001), and major bleeding (OR 1.44 95% CI 1.26–1.64, *p* < 0.001) but slightly lower regression coefficients for length of stay (coef 1.47 95% CI 1.34 to 1.60, *p* < 0.001) and cost (5342 95% CI 4684 to 6000, *p* < 0.001).

The evaluation of the impact of AF on outcomes according to use of adjustments and consideration of the category of the first diagnostic code is shown in Supplementary Table S2. The effects of adjustments reduced the magnitude of the association between AF and outcome, but all associations showed increased in adverse events, length of stay, and cost with AF. To provide further clarity on the reasons for hospitalization, we conducted additional sensitivity analyses, categorizing patients based on the primary diagnosis. These findings are illustrated in Supplementary Tables S3–S5.

## 4. Discussion

**Figure 2:**
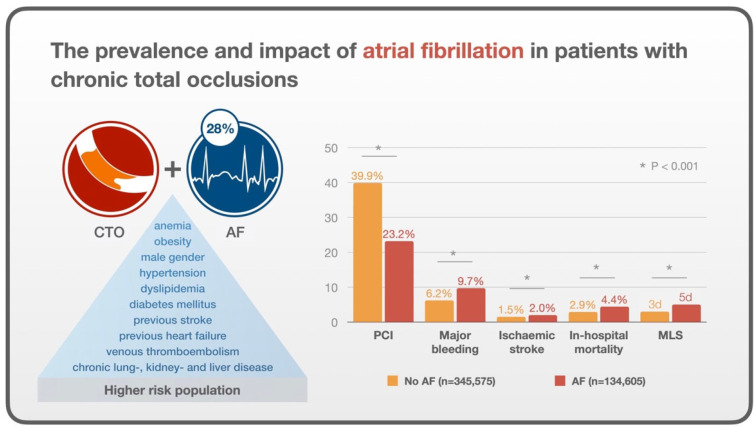
Central illustration: The prevalence and impact of atrial fibrillation in patients with chronic total occlusions.

Our study reports several key findings that are summarized in Figure 2. Firstly, patients who are hospitalized and found to have a diagnosis of CTO and concomitant AF were older and had more comorbidities as well as a greater rate of in-hospital ischemic stroke, major bleeding, and in-hospital mortality. Second, the greater comorbidity and poor outcomes are likely to have contributed to a greater length of stay and cost for the group with AF.

These findings suggest that patients with AF are a higher-risk group compared to patients without AF, and more studies are needed to know if AF itself a marker of risk or if it is the profile of the patient with AF that contributes to poor outcome. In addition, studies are needed to better understand the ideal management for patients with AF and CTO in order to minimize their risk, as these patients are at risk of ischemic stroke and bleeding, which may be related to antithrombotic use.

To the best of our knowledge, this is the first study to report the prevalence of AF in ‘all comer’ nationwide pool of patients with CTO, regardless of their further treatment path. Whereas two important RCTs [9,14] comparing revascularization versus optimal medical therapy in CTO are lacking information on the presence of AF, there are sparse registry data on the prevalence of AF in patients who underwent CTO PCI [11,12]. Alexandru et al. [11] report that 12% of patients with CTO PCI have AF too, which is comparable with a prior study of Staehli et al., who report a prevalence of 8.4% and with studies of patients undergoing non-CTO PCI [15,16]. A key finding in the current study is that it is more common than the rate reported in other studies, as more than 1 in 4 patients have AF.

The presence of AF in patients with CTOs in our cohort was associated with older age; male gender; obesity; hypertension; dyslipidemia; diabetes mellitus; previous stroke; previous heart failure and venous thromboembolism; chronic lung, kidney, and liver disease; and anemia. These common comorbidities associated with AF are widely recognized in the literature as contributors to the development of AF [17]. An important consideration is whether the AF directly contributes to poor outcomes in patients with CTO or if it is mediated by a more comorbid cohort, and it remains unclear the mechanisms for these poor outcomes. Evidently, our analysis revealed statistically significant differences across several variables. However, the clinical relevance of these differences requires careful consideration. For instance, the 0.8% difference in diabetes mellitus, while statistically significant, may have a negligible impact on clinical decision-making and patient outcomes in this setting. Similarly, the observed differences in the other variables, though notable, may not necessarily translate into meaningful changes in risk assessment or treatment interventions.

This cohort highlights the clinical complexity associated with AF, including frailty, multimorbidity, and polypharmacy [18,19,20]. Apart from stroke prevention and rhythm management, additional multidisciplinary preventive strategies, such as lifestyle modifications, might alleviate the burden of AF. This is aligned with current recommendations, for a holistic or integrated care approach to AF management [21], where improved clinical outcomes are seen by adherence to the Atrial fibrillation Better Care (ABC) pathway, including reductions in mortality, stroke, bleeding, and hospitalizations [22,23].

The treatment of AF with anticoagulation will shift the thrombotic-bleeding profile to that of increased risk of bleeding [24]. It is likely that the treatment of patients with CTO and AF needs to be individualized, with some having greater underlying bleeding risk where it may be acceptable to not have antithrombotic, while other patients have a greater thrombotic risk, thus supporting this use of anticoagulation [25,26].

The impact of AF on in-hospital outcomes following CTO PCI has been previously assessed by two studies, which found similar results. The first analyzed 2002 CTO PCIs performed in Germany between 2005 and 2013 and found higher periprocedural MACE with present AF (3.6% vs. 1.4%, *p* = 0.04) [12]. This finding was mainly driven by more frequently observed vascular access site complications and cardiac tamponade in patients with AF. The second study, by Alexandru et al. [11], showed no significant difference in procedural complications related to the presence of AF. One potential reason for the observed difference between both studies could be the growing utilization of radial access over time [26], given a decade gap between the analyzed time frames of the studies. Of note, the incidence of follow-up MACE and all-cause mortality was significantly higher in patients with AF in both studies. Our study supports these findings from a national American perspective but we were able to explore additional outcomes, such as ischemic stroke, bleeding, length of stay, and cost. How to minimize these adverse outcomes requires further studies.

In our cohort, AF among patients with CTOs was strongly associated with previous heart failure. This finding is aligned with Staehli et al. [12], reporting that AF was associated with reduced left ventricular systolic function in patients with CTOs. Mechanistically, AF can result in tachycardic or bradycardic left heart failure in patients with chronic total occlusions. However, patients with CTOs may also be more prone to develop heart failure as a consequence of increased left atrial pressure due to ischemia-related impairment of diastolic relaxation [3,26] or proarrhythmogenic scar tissue, raising a complex cause or effect question.

Our study suggests that major bleeding may play an important role in the high death rate among people with AF and CTOs. Naturally, when anticoagulation is required, bleeding complications are a logical concern. This risk is further heightened during PCI due to the potential for vascular access site complications, particularly with CTO-PCI [12].

### Limitations

Our study has several limitations. The NIS is an observational dataset with inherent biases, such as risk of bias from confounding. There was no independent adjudication of the diagnosis of AF and clinical events, and there was no core laboratory assessment of the study angiograms. Data on anticoagulant use and long-term bleeding data were not available, as well as information regarding detailed coronary anatomy, viability, and follow-up mortality or MACE.

The study population comprises 345,575 individuals without AF and 134,605 with AF. While numerous variables demonstrate statistical significance, the associated percentage differences are negligible. This phenomenon likely stems from the substantial sample size, which enables the detection of minor discrepancies as statistically meaningful. This represents an important limitation of the present investigation, necessitating cautious interpretation of the results.

## 5. Conclusions

Patients with coronary CTOs and concomitant AF were older and had more comorbidities in comparison to patients without AF in the observed cohort. Moreover, AF was associated with a higher risk of periprocedural complications, in-hospital mortality, longer median length of stay, and increased costs. Even though the study found statistically significant differences in several clinical factors, the broader clinical significance of these findings remains unclear. Further research is warranted to determine whether CTO lesions have wider clinical relevance in patients with AF.

## Figures and Tables

**Table 1:** Characteristics of patients with atrial fibrillation according to presence of chronic total occlusion.

Variable	No AF (n = 345,575)	AF (n = 134,605)	*p*-Value
Median age [IQR]	66 [58 to 74]	73 [66 to 80]	<0.001
Female	27.9%	25.0%	<0.001
Smoking	1.4%	0.9%	<0.001
Alcohol misuse	1.8%	1.5%	0.014
Obesity	21.5%	21.9%	0.15
Hypertension	86.9%	90.7%	<0.001
Hypercholesterolaemia	76.4%	75.6%	0.006
Diabetes mellitus	48.5%	47.7%	0.023
Previous myocardial infarction	29.6%	31.5%	<0.001
Previous stroke	11.5%	14.1%	<0.001
Previous heart failure	28.8%	48.4%	<0.001
Previous venous thromboembolism	3.9%	5.0%	<0.001
Chronic lung disease	24.7%	31.4%	<0.001
Chronic kidney disease	26.0%	37.5%	<0.001
Liver disease	3.9%	5.1%	<0.001
Anemia	7.8%	10.2%	<0.001
Cancer	3.6%	5.0%	<0.001
Dementia	3.1%	4.7%	<0.001
Major bleeding	6.2%	9.7%	<0.001
Percutaneous coronary intervention	39.9%	23.2%	<0.001
Ischemic stroke	1.5%	2.0%	<0.001
In-hospital mortality	2.9%	4.4%	<0.001
Median length of stay [IQR]	3 [2 to 6]	5 [3 to 9]	<0.001
Median hospital cost [IQR]	USD 18,747 [11,252 to 31,695]	USD 21,671 [11,148 to 40,884]	<0.001

AF = atrial fibrillation; IQR = interquartile range.

**Table 2:** Predictors of atrial fibrillation in patients with chronic total occlusions (n = 454,110).

Variable	Odds Ratio (95% CI)	*p*-Value
Age	1.05 (1.04–1.05)	<0.001
Female sex	0.75 (0.73–0.78)	<0.001
Race vs. White		
Black	0.59 (0.55–0.62)	<0.001
Hispanic	0.62 (0.58–0.66)	<0.001
Asian or Pacific Islander	0.81 (0.73–0.89)	<0.001
Native American	0.60 (0.48–0.75)	<0.001
Other	0.69 (0.63–0.76)	<0.001
Smoking	0.85 (0.73–0.99)	0.042
Alcohol misuse	1.15 (1.01–1.130)	0.029
Primary expected payer vs. Medicare		
Medicaid	0.82 (0.77–0.88)	<0.001
Private insurance	0.91 (0.87–0.95)	<0.001
Self-pay	0.74 (0.66–0.83)	<0.001
ZIP income quartile vs. 0th–25th		
51st–75th	1.05 (1.00–1.10)	0.039
76th–100th	1.08 (1.03–1.13)	0.002
Obesity	1.25 (1.20–1.30)	<0.001
Hypertension	1.17 (1.11–1.23)	<0.001
Diabetes mellitus	0.92 (0.84–0.90)	<0.001
Previous stroke	1.15 (0.89–0.95)	<0.001
Previous heart failure	1.98 (1.92–2.05)	<0.001
Previous venous thromboembolism	1.18 (1.09–1.27)	<0.001
Chronic lung disease	1.18 (1.14–1.23)	<0.001
Chronic kidney disease	1.20 (1.16–1.24)	<0.001
Liver disease	1.37 (1.27–1.48)	<0.001
Dementia	0.92 (0.85–0.99)	0.036
Anemia	1.16 (1.10–1.22)	<0.001

**Table 3:** Multivariable outcomes for patients with chronic total occlusion and atrial fibrillation.

Impact of AF on Outcomes for Patient with CTO			
Outcome	n	Odds ratio (95% CI)	*p*-value
In-hospital mortality	454,110	1.29 (1.18–1.40)	<0.001
Ischemic stroke	454,110	1.27 (1.13–1.42)	<0.001
Major bleeding	454,110	1.38 (1.30–1.46)	<0.001
**Outcome**	**n**	**Coefficient [95% CI)**	***p*-value**
Length of stay	454,110	1.58 [1.50 to 1.67]	<0.001
Cost	450,800	6219 [5811 to 6627]	<0.001
**Impact of AF on Outcomes for Patient with CTO in Patients with PCI**			
**Outcome**	**n**	**Odds ratio (95% CI)**	***p*-value**
In-hospital mortality	159,525	1.55 (1.35–1.78)	<0.001
Ischemic stroke	159,525	1.53 (1.19–1.96)	0.001
Major bleeding	159,525	1.44 (1.26–1.64)	<0.001
**Outcome**	**n**	**Coefficient [95% CI)**	***p*-value**
Length of stay	159,525	1.47 (1.34–1.60)	<0.001
Cost	158,340	5342 [4684 to 6000)	<0.001

## Data Availability

The data used in this study were obtained from the National Inpatient Sample (NIS), which is part of the Healthcare Cost and Utilization Project (HCUP) maintained by the Agency for Healthcare Research and Quality (AHRQ). The NIS dataset is available for purchase and access through HCUP’s online database.

## Supplementary Materials

The following supporting information can be downloaded at: https://www.mdpi.com/article/10.3390/jcdd12030100/s1, Table S1: Description of codes to the data analysis; Table S2: Impact of atrial fibrillation on outcomes for patients with chronic total occlusion according to level of adjustments, Table S3: Cause of admission according to primary diagnostic code, Table S4: Cause of admission sensitivity analysis, Table S5: Primary diagnosis of acute myocardial infarction sensitivity analysis.
