# Supplementary material for: The Prevalence and Impact of Atrial Fibrillation on Patients with Chronic Total Occlusions: Insights from the National Inpatient Sample
Source: J Cardiovasc Dev Dis. 2025 Mar 14;12(3):100. doi: 10.3390/jcdd12030100 (PMC11943249; doi:10.3390/jcdd12030100)
Supplement: Supplementary file 1 [file jcdd-12-00100-s001.zip › jcdd-3474671-supplementary.pdf]

**Supplementary Files**

**Supplementary Table S1.** Description of codes to the data analysis

| Variable | Source | ICD-10 code |
|---|---|---|
| Atrial fibrillation or flutter | I10_DX1/40 | I48* |
| Chronic total occlusion | I10_DX1/40 | I25.82 |
| Age | NIS Core | AGE |
| Female sex | NIS Core | FEMALE |
| Race | NIS Core | RACE |
| Smoking (tobacco use) | I10_DX1/40 | Z72.0 |
| Alcohol misuse | I10_DX1/40 | F10.1 |
| Elective admission | NIS Core | ELECTIVE |
| Weekend admission | NIS Core | AWEEKEND |
| Season | NIS Core | AMONTH Spring = March to May, Summer = June to Aug, Fall = Sept to Nov, Winter = Dec to Feb |
| Rural hospital | NIS Core | PL_NCHS |
| Hospital bed size | NIS Hospital | HOSP_BEDSIZE |
| Primary expected payer | NIS Core | PAY1 |
| ZIP income quartile | NIS Core | ZIPINC_QRTL |
| Obesity | I10_DX1/40 | E66.0, E66.1, E66.2, E66.8, E66.9 |
| Hypertension | I10_DX1/40 | I10*, I11*, I12*, I13*, I15*, I16* |
| Hyperlipidemia | I10_DX1/40 | E78.0*, E78.1, E78.2, E78.3, E78.4*, E78.5 |
| Diabetes mellitus | I10_DX1/40 | E08*, E09*, E10*, E11*, E13* |
| Previous myocardial infarction | I10_DX1/40 | I25.2 |
| Previous stroke | I10_DX1/40 | Z86.73, I69* |
| Previous heart failure | I10_DX1/40 | I50.22, I50.23, I50.32, I50.33, I50.42, I50.43, I50.812, I50.813 |
| Previous venous thrombosis/embolism | I10_DX1/40 | Z86.71 |
| Chronic lung disease | I10_DX1/40 | J40* – J47* |
| Chronic kidney disease | I10_DX1/40 | N18* |
| Liver diseases | I10_DX1/40 | K70-K77 |
| Anemia | I10_DX1/40 | D64.9 |
| Any cancer | I10_DX1/40 | C00*-C96* |
| Dementia | I10_DX1/40 | F01*, F02*, F03*, G30*, G31* |
| Major bleeding | I10_DX1/40<br><br><br><br><br><br>I10_PR1/25 | I60*, I61*, I62*, I69.0, I69.1, I69.2, K92.0, K92.1, K92.2, K25.0, K25.1, K25.2, K25.4, K25.5, K25.6, K26.0, K26.1, K26.2, K27.0, K27.1, K27.2, K27.4, K27.5, K27.6, K28.0, K28.1, K28.2, K28.4, K28.5, K28.6<br>30243N0, 30243N1, 30243P0, 30243P1, 30243H0, 30243H1, |

| | | |
|---|---|---|
| | | 30240N0, 30240N1, 30240P0, 30240P1, 30240H0, 30240H1, 30230H0, 30230N0, 30230N1, 30230P0, 30230P1, 30233N0, 30233N1, 30233P0, 30233P |
| Percutaneous coronary intervention | I10_PR1/25 | 02703D6, 02703DZ, 02704D6, 02704DZ, 02703E6, 02703EZ, 02704E6, 02704EZ, 02703F6, 02703FZ, 02704F6, 02704FZ, 02703G6, 02703GZ, 02704G6, 02704GZ, 02713D6, 02713DZ, 02714D6, 02714DZ, 02713E6, 02713EZ, 02714E6, 02714EZ, 02713F6, 02713FZ, 02714F6, 02714FZ, 02713G6, 02713GZ, 02714G6, 02714GZ, 02723D6, 02723DZ, 02724D6, 02724DZ, 02723E6, 02723EZ, 02724E6, 02724E6, 02724EZ, 02723F6, 02723FZ, 02724F6, 02724FZ, 02723G6, 02723GZ, 02724G6, 02724GZ, 02733D6, 02733DZ, 02734D6, 02734DZ, 02733E6, 02733EZ, 02734E6, 02734EZ, 02733F6, 02733FZ, 02733FZ, 02734F6, 02733G6, 02733GZ, 02734G6, 02734GZ, 0270346, 027034Z, 0270446, 027044Z, 0270356, 027035Z, 0270456, 027045Z, 0270366, 027036Z, 0270466, 027046Z, 0270376, 027037Z, 0270476, 027047Z, 0271346, 027134Z, 0271446, 027144Z, 0271356, 027135Z, 0271456, 027145Z, 0271366, 027136Z, 0270376, 0271466, 027146Z, 0271376, 027137Z, 0271476, 027147Z, 0272346, 027234Z, 0272446, 027244Z, 0272356, 027235Z, 0272456, 027245Z, 0272366, 027236Z, 027246Z, 0272376, 027237Z, 0272476, 027035Z, 027247Z, 0273346, 027334Z, 0273446, 027344Z, 0273356, 027335Z, 0273456, 027345Z, 0273366, 027336Z, 0273466, 027346Z, 0273376, 027337Z, 027045Z, 0273476, 027347Z, 02703ZZ, 02704ZZ, 02713ZZ, 02714ZZ, |

| | | |
|---|---|---|
| | | 02723ZZ, 02724ZZ, 02733ZZ, 02734ZZ |
| Acute ischemic stroke | I10_DX1/40 | I63* |
| Discharge weight | NIS Core | DISCWT |
| Discharge disposition | NIS Core | DISPUNIFORM |
| In-hospital mortality | NIS Core | DIED |
| Length of stay | NIS Core | LOS |
| Total charge | NIS Core | TOTCHG |
| Cost | - | Total charge x charge-to-cost ratio |

**Supplementary Table S2.** Impact of atrial fibrillation on outcomes for patients with chronic total occlusion according to level of adjustments

| Outcome | Unadjusted model | Adjusted for all baseline variables and comorbidities | Adjusted for all baseline variables, comorbidities and primary diagnosis category |
|---|---|---|---|
| In-hospital mortality | OR 1.52 (1.42-1.64), p<0.001 | OR 1.29 (1.18-1.40), p<0.001 | OR 1.28 (1.18-1.39), p<0.001 |
| Ischemic stroke | OR 1.33 (1.20-1.48), p<0.001 | OR 1.27 (1.13-1.42), p<0.001 | OR 1.23 (1.10-1.38), p<0.001 |
| Major bleeding | OR 1.63 (1.55-1.71), p<0.001 | OR 1.38 (1.30 to 1.46), p<0.001 | OR 1.40 (1.32-1.48), p<0.001 |
| Length of stay | Coefficient 2.11 [2.03 to 2.19], p<0.001 | Coefficient 1.58 [1.50 to 1.67], p<0.001 | Coefficient 1.57 [1.49 to 1.66], p<0.001 |
| Cost | Coefficient 6156 [5769 to 6544], p<0.001 | Coefficient 6219 [5811 to 6627], p<0.001 | Coefficient 6125 [5718 to 6532], p<0.001 |

**Supplementary Table S3**. Cause of admission according to primary diagnostic code

| Category for the primary diagnosis code | % total |
|---|---|
| Diseases of the circulatory system (I) | 82.7 |
| Injury, poisoning and certain other consequences of external cause (S) | 4.3 |
| Diseases of the respiratory system (J) | 2.4 |
| Infectious and parasitic diseases (A) | 2.1 |
| Symptoms, signs and abnormal clinical or laboratory findings not elsewhere classified (R) | 2.0 |
| Diseases of the digestive system (K) | 1.8 |
| Endocrine, nutritional and metabolic diseases (E) | 1.1 |
| Other | 3.5 |

**Supplementary Table S4.** Cause of admission sensitivity analysis

| Primary diagnosis or diagnosis category | n | % of total | % with AF | Mortality without AF (%) | Mortality with AF (%) |
|---|---|---|---|---|---|
| Chronic ischemic heart disease (I25) | 152730 | 31.8 | 24.0 | 0.8 | 1.5 |
| Acute myocardial infarction (I21) | 135890 | 28.3 | 19.3 | 4.5 | 8.1 |
| Hypertensive heart and renal disease (I13) | 21510 | 4.5 | 44.4 | 3.2 | 4.0 |
| Hypertensive heart disease (I11) | 15900 | 3.3 | 34.8 | 1.2 | 1.6 |
| Heart failure (I50) | 11400 | 2.4 | 37.1 | 2.9 | 4.3 |
| Paroxysmal tachycardia (I47) | 10725 | 2.2 | 40.8 | 4.1 | 3.9 |
| Atrial fibrillation and flutter (I48) | 8880 | 1.8 | 100 | 1.5 | 0.9 |
| Non-rheumatic aortic valve disorder (I35) | 8290 | 1.7 | 43.5 | 5.0 | 3.2 |
| Other circulatory causes | 31605 | 6.6 | 32.2 | 2.7 | 6.4 |
| Injury, poisoning and certain other consequences of external cause (S) | 20780 | 4.3 | 27.2 | 5.9 | 4.9 |
| Diseases of the respiratory system (J) | 11660 | 2.4 | 35.9 | 10.1 | 8.2 |
| Infectious and parasitic diseases (A) | 10255 | 2.1 | 35.0 | 1.7 | 12.3 |
| Symptoms, signs and abnormal clinical or laboratory findings not elsewhere classified (R) | 9675 | 2.0 | 24.8 | 2.6 | 2.5 |
| Diseases of the digestive system (K) | 8700 | 1.8 | 35.3 | 0.9 | 5.4 |
| Endocrine, nutritional and metabolic diseases (E) | 5335 | 1.1 | 25.6 | 2.6 | 2.2 |
| Chronic ischemic heart disease | 16845 | 3.5 | 31.2 | 2.9 | 4.1 |

**Supplementary Table S5.** – Primary diagnosis of acute myocardial infarction sensitivity analysis

| Primary diagnosis | n | % with AF | Mortality without AF | Mortality with AF |
|---|---|---|---|---|
| NSTEMI (I21.4) | 88250 | 21.6 | 2.5 | 5.1 |
| STEMI (I21.0, I21.1, I21.2, I21.3) | 38700 | 15.0 | 7.4 | 15.3 |
| HF (I50) | 11400 | 37.1 | 2.9 | 4.3 |
| HF no support | 10860 | 37.4 | 2.1 | 2.7 |
| HF IABP (5A02210) | 310 | 30.7 | 16.3 | 42.1 |
| HF LVAD (5A02116, 5A0211D, 5A02216, 5A0221D) | 270 | 31.5 | 16.2 | 47.1 |
